# Low-Power Field-Deployable Interdigital Transducer-Based Scanning Laser Doppler Vibrometer for Wall-Thinning Detection in Plates

**DOI:** 10.3390/ma17205098

**Published:** 2024-10-18

**Authors:** To Kang, Soonwoo Han, Yun-Taek Yeom, Ho-Yong Lee

**Affiliations:** 1 Korea Atomic Energy Research Institute, Daejeon 34057, Republic of Korea; tkang@kaeri.re.kr (T.K.); swhan@kaeri.re.kr (S.H.); 2 Department of Smart Mechanical Engineering, Dongyang University, Yeongju 36040, Republic of Korea; 3 Ceracomp Co., Ltd., Cheonan 31094, Republic of Korea; hlee.ceracomp@gmail.com

**Keywords:** Lamb wave, low-power interdigital transducer, scanning laser Doppler vibrometer, wavenumber sensitivity, thin-wall plate detection

## Abstract

Lamb waves have become a focal point in ultrasonic testing owing to their potential for long-range and inaccessible detection. However, accurately estimating the flaws in plates using Lamb waves remains challenging because of scattering, mode conversion, and dispersion effects. Recent advances in laser ultrasonic wave techniques have introduced innovative visualization methods that exploit the dispersion effect of Lamb waves to visualize defects via, for example, acoustic wavenumber spectroscopy. In this study, we developed an interdigital transducer (IDT)-based scanning laser Doppler vibrometer (SLDV) system without a power amplifier using a low-power IDT fabricated from lead magnesium niobate–lead zirconate titanate single crystals. To validate the proposed low-power IDT-based SLDV, four different defective plates were measured for defects. A comparison between a conventional IDT-based SLDV, a dry-coupled IDT-based SLDV, and the proposed method demonstrated that the latter is highly reliable for measuring thin plate defects.

## 1. Introduction

Lamb waves have garnered significant interest from researchers owing to their capacity to be used in inaccessible or long-range detection applications. These waves offer a unique advantage in ultrasonic testing by providing information about specimens from scattered waves, which is challenging to achieve via traditional methods. However, the data obtained from Lamb waves regarding specimen defects, particularly from scattered waves, are often insufficient for accurately estimating the defects in plates. Consequently, many studies have aimed to quantitatively analyze Lamb wave properties, including reflection, mode conversion, and the interactions between symmetric, anti-symmetric, and shear horizontal waves [1,2,3,4]. However, the complex behavior of these waves, which is characterized by scattering, diffraction, and mode conversion, poses significant challenges in detecting minor pitting defects or gradual wall thinning. Additionally, the dispersion effect, which results in the spreading of Lamb waves over time and distance as they traverse a plate, is a critical issue [5,6,7,8].

In response to these challenges, laser ultrasonic wave techniques have been adopted [9,10,11,12,13,14,15,16,17,18,19,20,21,22,23,24,25,26,27,28] to enable defects to be visualized through signal processing methods without the need for intricate Lamb wave analysis. Quantitative visualization imaging algorithms such as acoustic wavenumber spectroscopy (AWS) [18], local wavenumber domain estimation [15,29,30,31], local wavenumber mapping [32,33], and 2D wavenumber estimation [34] have been employed. Moreover, the dispersion effect of Lamb waves, which has been traditionally viewed as a drawback because of its impact on wave propagation, is used in these visualization methods. Specifically, the phase velocity changes in Lamb waves passing through structures provide valuable thickness information, transforming the dispersion effect from a disadvantage into a useful tool for quantitatively assessing structures.

Previous studies on the use of exciters and receivers for ultrasonic wave generation and detection in plates have explored several combinations, including pulsed lasers, piezoelectric transducers (PZTs), and air-coupled transducers used as exciters and laser Doppler vibrometers, PZTs, or air-coupled transducers used as receivers. Notable work on the optimization of exciter and receiver configurations was conducted by An et al. [10], who identified that the combination of scanning laser excitation with fixed-point PZT sensing yielded the highest signal-to-noise ratio among the laser ultrasonic scanning setups explored, which included fixed-point PZT excitation with scanning laser sensing, scanning laser excitation with fixed-point PZT sensing, fixed-point laser excitation with scanning laser sensing, and scanning laser excitation with fixed-point laser sensing. Their research primarily focused on the analysis of propagated and scattered waves from defects in a plate. Addressing a different aspect, Flynn and Jarmer introduced a method for the high-speed imaging of plate defects using steady-state ultrasonic vibration excited by a continuous sinusoidal wave [35]. This approach, particularly the fixed-point PZT excitation with a scanning laser sensing configuration, allowed for high energy acquisition owing to energy efficiently pumping into the structures, the absence of measurement delays, and reduced acquisition times. This was achieved while ensuring operational safety using class 1–2 lasers with lower power compared to those used in propagation wave analysis methods. Consequently, continuous-based excitation using scanning laser Doppler vibrometers (SLDVs) has emerged as a powerful tool for visualizing and detecting defects in plates.

Despite the advantages of SLDVs over scanning laser excitation methods, using the A_0_ Lamb wave mode complicates the detection of shallow wall thinning in plates. Early damage detection is crucial for preventing catastrophic failures in industrial facilities, such as nuclear and thermal power plants. Traditional SLDV approaches, which use A_0_ mode for wall-thinning detection, must contend with the mode’s dispersive nature, which impacts the accuracy of evaluating the thickness of wall thinning. This limitation restricts the wall-thinning detection capability of SLDV to thicknesses higher than 30% in plates and pipes. To address this challenge, Kang et al. [36,37] and Moon et al. [38] explored the detection of shallow wall thinning using an interdigital transducer (IDT)-based SLDV [36,37,38], which offers a promising avenue for enhancing early damage detection capabilities.

IDTs are manufactured using a picosecond laser machining process and are engineered to excite modes sensitive to shallow wall thinning, which are characterized by a high wavenumber sensitivity. In the studies of Kang et al. and Moon et al. [36,37,38], IDT-based SLDVs were proven capable of detecting wall thinning higher than 5%, aligning with the requirements of KEPIC 3521 for nuclear power plants. A notable limitation of both PZT and IDT technologies is their dependence on a couplant, such as oil or water, which complicates and encumbers the inspection process. To address this, Kang et al. [39] introduced a dry-coupled IDT-based SLDV system. This innovative approach used a λ/4 impedance transformer with alumina for dry coupling and successfully detected wall thinning over 3% in plates without requiring any couplant.

Despite these advances, and even though the dry-coupled IDT-based SLDV system is more amenable to field deployment, it still faces challenges that need resolution. One significant issue is the power consumption of the exciter. Typically, a power amplifier is used to enable the IDT to generate a high-power sinusoidal wave in IDT-based SLDV operations. For instance, the HSA4052 model from the NF Corporation(Yokohama, Japan), with its maximum voltage of ±75 V at 25 Ω, is commonly used.

In this study, we propose a novel methodology for operating the IDT-based SLDV without a power amplifier. This approach involves replacing the conventional IDT material with a CSL2 (PMN-PZT) to enhance the sensitivity of IDT sensors, thereby developing a highly reliable system that does not require a power amplifier. The rest of this paper is structured as follows. Section 2 describes the development of the newly designed low-power IDT-based SLDV. Section 3 evaluates the performance of the low-power IDT-based SLDV through a comparative analysis; it also elaborates on the performance metrics of the IDT-based SLDV, dry-coupled IDT-based SLDV, and low-power IDT-based SLDV, highlighting their differences and capabilities. Section 4 concludes the paper with a summary that encapsulates the findings and potential implications of this research.

## 2. Low-Power IDT-Based SLDV

### 2.1. Fabrication of Lead Magnesium Niobate–Lead Zirconate Titanate Single Crystals

**Figure 1:**
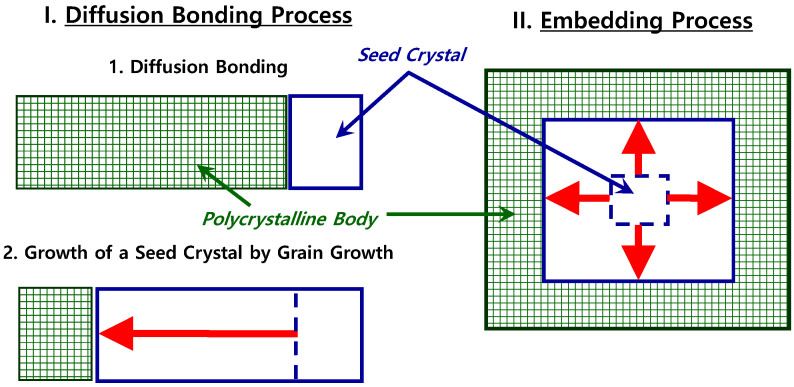
Solid-state single crystal growth (SSCG) method: illustration of single crystal development within a polycrystalline matrix via grain growth.

In this study, the solid-state single crystal growth (SSCG) method was used to synthesize lead magnesium niobate–lead zirconate titanate (PMN-PZT) [Pb(Mg_1/3_Nb_2/3_)O_3_-Pb(Zr,Ti)O_3_] piezoelectric single crystals (Product Code: CSL2) at Ceracomp Co., Ltd. (Cheonan, Republic of Korea) [40,41,42,43,44]. Figure 1 displays a schematic representation of the SSCG method. 

Among the various kinds of PMN-PZT single crystals, the PMN-PZT (CSL2) single crystal was selected because it has high piezoelectric constants, has a composition of “40PMN-25PZ-35PT + 1.0La_2_O_3_ [mol%]”, and exhibits a rhombohedral phase at room temperature. Compared to conventional single-crystal methods, such as the flux and Bridgman methods, the SSCG method is more cost-effective and suitable for mass production because the difficult steps of melting and solidification in the conventional methods can be completely avoided in the SSCG process. Because of its advantages, the SSCG method is the most effective way of growing high-performance piezoelectric single crystals with very complicated chemical compositions.

**Figure 2:**
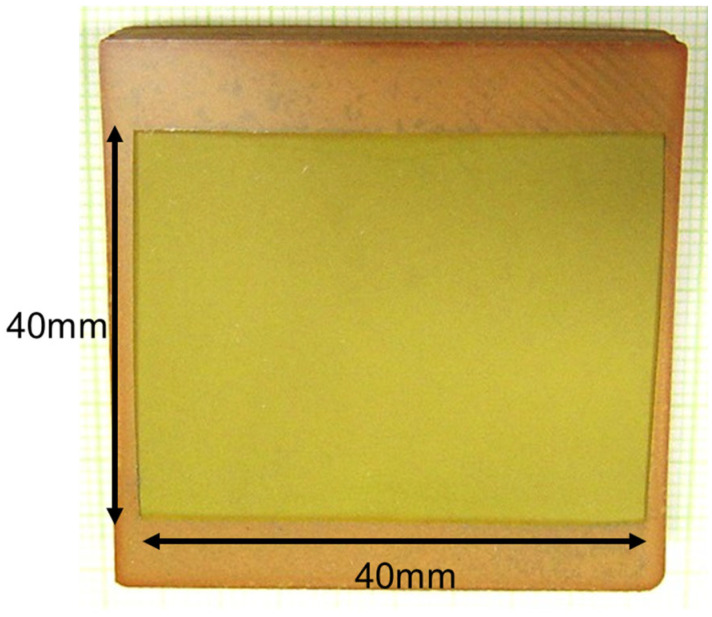
Image of PMN-PZT piezoelectric single crystal grown in polycrystalline ceramics via the SSCG method.

High-purity raw materials, including Pb_3_O_4_ (99.9%, Alfa Aesar, Ward Hill, MA, USA), MgNb_2_O_6_ (99.9%, H. C. Starck GmbH, Newton, MA, USA), and TiO_2_ (99.99%, Ishihara, San Francisco, CA, USA), were selected for the fabrication process. After precisely measuring each raw material, the powders were subjected to ball milling for 24 h, then dried and calcined at 800 °C. The calcinated powders were further processed via secondary ball milling, with the addition of excess PbO powder, and were subsequently dried and sieved to produce the final powder. This powder was then uniaxially hot pressed at high temperatures to form a dense, primary sintered body. For the SSCG process, a Ba(Zr_0.1_Ti_0.9_)O_3_ seed single crystal was positioned on top of the ceramic sintered body, after which a specialized heat treatment was performed. To mitigate the loss of volatile PbO, the double-crucible method was used during the SSCG heat treatment. This process facilitated the continuous growth of the Ba(Zr_0.1_Ti_0.9_)O_3_ seed single crystal within the polycrystalline ceramic matrix, culminating in the production of single crystals measuring 40 mm × 40 mm × 10 mm, as shown in Figure 2. A notable advantage of the SSCG method is the absence of composition gradients within the resulting single crystal, which ensures chemical uniformity. This uniformity is attributed to the fact that the PMN-PZT phase does not melt during the fabrication process, thereby preserving the integrity of the single crystal structure. 

Figure 2 shows a photograph of a PMN-PZT piezoelectric single crystal produced using the SSCG method.

### 2.2. Design and Fabrication of Low-Power IDT

In linear piezoelectric materials, the interactions between electrical and mechanical variables conform to linear relationships as defined by the ANSI/IEEE Standard 176-1987 [45]. The constitutive relationships that govern the interaction between these variables are

(1) $$S=s^{E}T+d$$

(2) $$D=dT+\varepsilon^{T}E$$

where $s^{E}$ is the elastic compliance under a constant electric field, T is the stress, d is the piezoelectric charge constant, E is the electric field, S is the strain, $\varepsilon^{T}$ is the permittivity of the ceramic material, and D is the electric flux density. An increase in the strain necessitates a corresponding increase in the elastic compliance, stress, piezoelectric charge constant, or the electric field. However, given that $s^{E}$, T, and E remain constant, the variable *d* becomes the focal point for adjustment.

Table 1 compares the performance metrics of the developed PMN-PZT (CSL2) single crystals with those of APC 850 (PZT) polycrystalline ceramics. CSL2 was categorized into two subtypes based on the crystallographic orientation: CSL2 (001) exhibited higher *k*_33_ and *d*_33_ constants (appropriate for the longitudinal vibration mode), and CSL2 (011) exhibited higher *k*_31_ and *d*_31_ constants (appropriate for the lateral vibration mode).

In Table 1, tanδ is the dielectric loss, $k_{ij}$ is the electromechanical coupling factor, *i* represents the direction of the electric field, and j represents the direction of the vibration. The term $d_{ij}$ corresponds to the piezoelectric charge constant previously mentioned, where polarization is induced in direction i (aligned with the piezoelectric element’s polarization) per unit stress applied in direction j (perpendicular to the ceramic element’s polarization) [46,47].

The CSL2 (001) subtype, operating in a longitudinal vibration mode, exhibited a $d_{33}$ value of 1952 pC/N, significantly surpassing APC 850’s $d_{33}$ value of 400 pC/N. Similarly, the CSL2 (011) subtype in the lateral vibration mode had a $d_{31}$ value of 919 pC/N, compared to APC 850’s $d_{31}$ value of 175 pC/N. Specifically, the $d_{33}$ value for CSL2 (001) was 4.88 times higher than that for APC 850. In addition, the $d_{31}$ value for CSL2 (011) was 5.25 times higher than that for APC 850. These comparisons confirmed CSL2’s suitability as an IDT material for low-power applications owing to its superior $d_{33}$ and $d_{31}$ values relative to PZTs.

**Figure 3:**
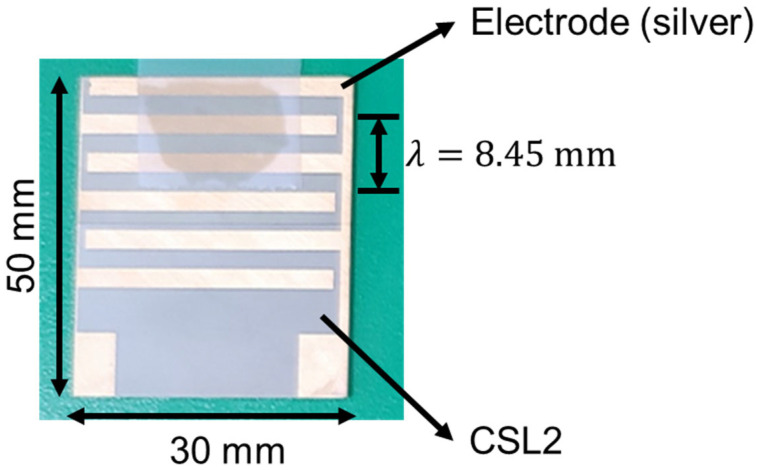
Prototype of low-power interdigital transducer (IDT) using CSL2.

Figure 3 shows the low-power IDT produced using CSL2.

**Figure 4:**
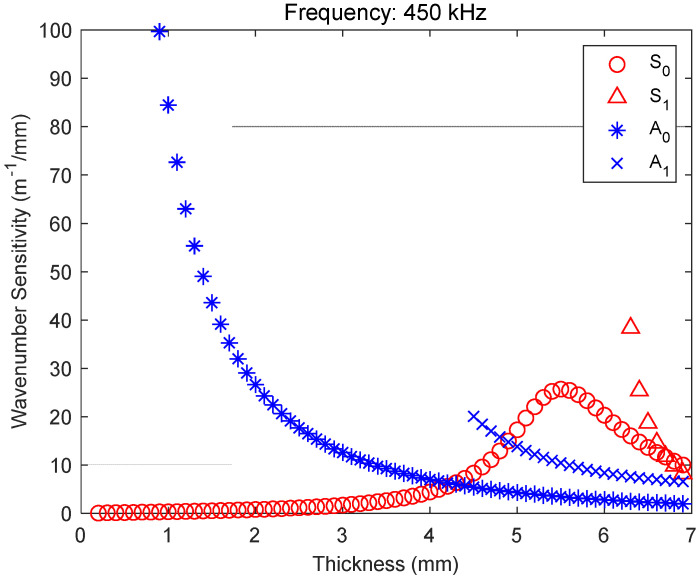
Wavenumber sensitivity at 450 kHz for carbon steel.

Based on previous studies [36,37,38,39], the optimal frequency for the low-power IDT was determined, and the wavenumber sensitivity for 450 kHz was obtained by taking the derivative of the dispersion curve, as shown in Figure 4.

The mode of $A_{0}$ exhibited a favorable sensitivity for thicknesses of less than 2 mm but was unsuitable for plates with a thickness of 6 mm. In contrast, the $S_{0}$ mode exhibited a high sensitivity in the plates with a thickness of 6 mm, which demonstrates the precise design for efficient mode generation in low-power IDTs.

**Figure 5:**
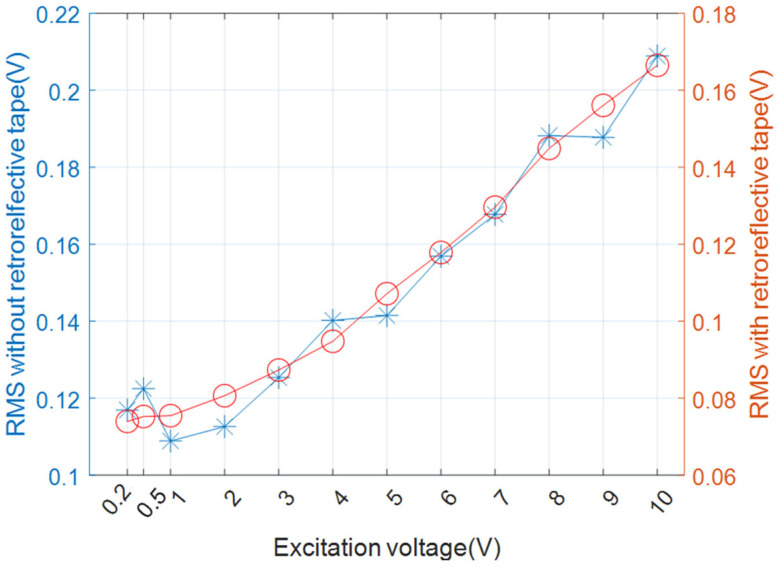
RMS as a function of excitation voltage for the $d_{33}$-type CSL2 with (orange circles) and without (blue stars) retroreflective tape.

To assess the performance of the fabricated low-power IDT, the RMS value was measured under several excitation voltages. In this study, CSL2 (001) is referred to as the $d_{33}$-type CSL2, and CSL2 (011) as the $d_{31}$-type CSL2. The relationship between the RMS value and the excitation voltage was linear. Using retroreflective tape enhanced the stability of the optical measurements, resulting in more stable RMS values compared to measurements taken without the tape [44]. Figure 5 illustrates the RMS measurements for the $d_{33}$-type CSL2, comparing scenarios with and without the retroreflective tape.

**Figure 6:**
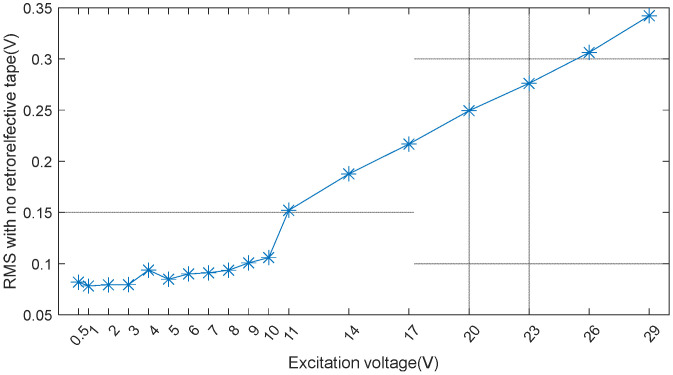
RMS as a function of excitation voltage for the $d_{31}$-type CSL2 (without retroreflective tape).

The results indicated that the RMS values consistently exceeded 0.2 V (irrespective of the tape’s presence) at an excitation voltage of 10 V. This suggests that the device can operate effectively without the need for a power amplifier. Figure 6 shows the RMS values for the $d_{31}$-type CSL2, for which tests were conducted exclusively without retroreflective tape.

The findings demonstrated that a minimum excitation voltage of approximately 15–16 V was necessary to achieve RMS values above 0.2 V. Given that commercial function generators typically provide 10 V or less, this limitation underscores the necessity for a power amplifier for the $d_{31}$-type CSL2.

A comparative analysis of Figure 5 and Figure 6 revealed that the $d_{33}$-type CSL2 exhibited a more efficient performance than the $d_{31}$-type CSL2. Considering the 10 V limit of commercial function generators, the $d_{33}$-type CSL2 was selected as the preferred material for low-power IDT applications.

## 3. Experiment

### 3.1. Wall-Thinning Measurement Procedure Using AWS

**Figure 7:**
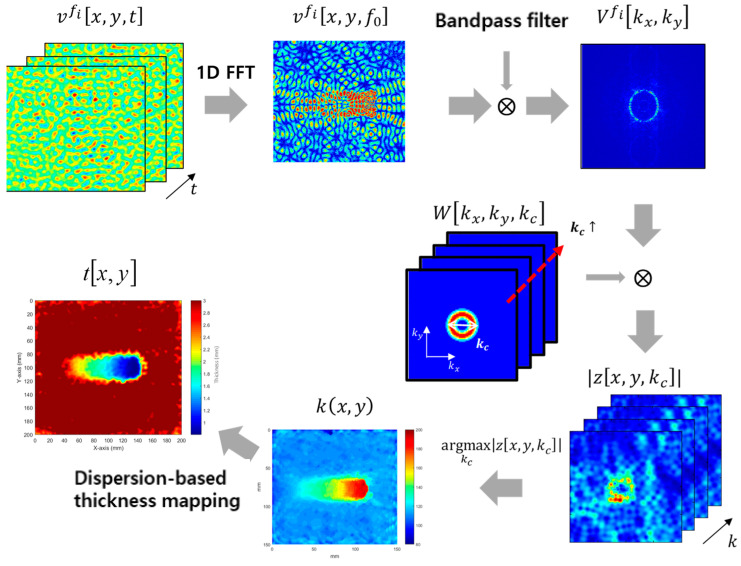
Procedure for measuring the thickness of plates using modified AWS.

The modified AWS technique, as proposed by Kang et al. [37], was used to reconstruct the thickness of a plate. This modified approach builds upon the original AWS method developed by Flynn and Jarmer [35], incorporating adjustments to enhance image quality with captured signals. The procedure of the modified AWS method, which is depicted in Figure 7, emphasizes the importance of the bandpass filter in eliminating unwanted propagation modes in $v^{f_{i}}[x,y,f_{0}]$. As the cutoff frequency increases, a continuous augmentation occurs in the number of modes in the high-frequency thickness regime, indicating that the bandpass filter is essential for isolating pure propagation modes. A key innovation of this modified AWS method compared to the original method is dispersion-based thickness mapping. The fusion of images at different frequencies allows for the accurate visualization of defects at varying depths, overcoming the limitations of traditional single-mode approaches and providing intuitive thickness information that facilitates the inspection process [37].

### 3.2. Specimen and Experimental Setup

**Figure 8:**
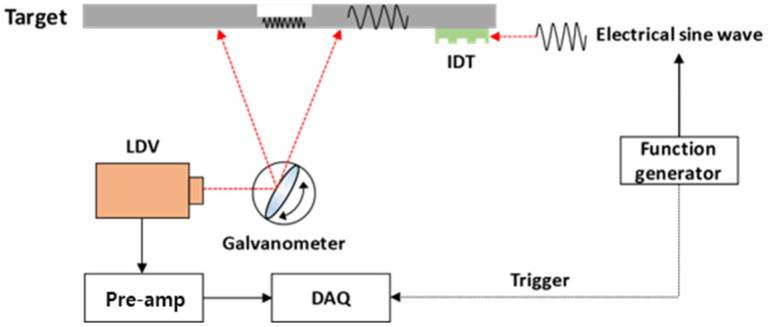
Schematic of low-power IDT scanning laser Doppler vibrometer (SLDV).

Figure 8 illustrates the method for measuring the thickness of a thin-wall plate with a low-powered IDT scanning laser Doppler vibrometer (SLDV).

**Figure 9:**
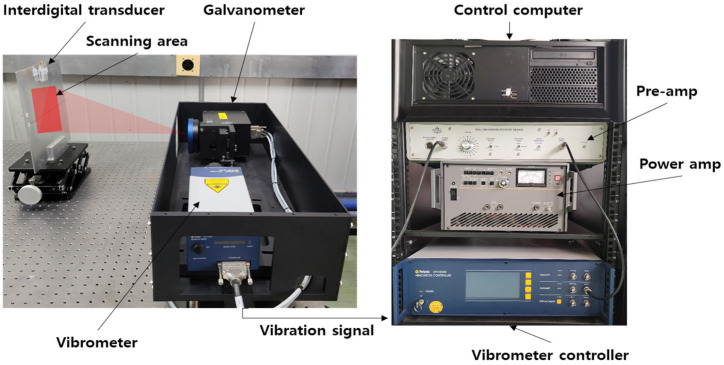
Setup for scanning laser Doppler vibrometer (SLDV) experiments.

To validate the functionality of the low-power IDT, the AWS method was used in the experimental setup. A function generator (PXI-5402) was used to produce a single-frequency sine wave, a high-speed bipolar amplifier (HSA4052) was used to deliver a high-voltage signal, and three types (standard, dry-coupled, and low-power) of IDTs were used to generate excitations. A scan head unit was used for the scanning vibrometer, which received the signal, and a data acquisition board (PCI-5124) recorded the vibration signals. The experimental arrangement is depicted in Figure 9.

**Figure 10:**
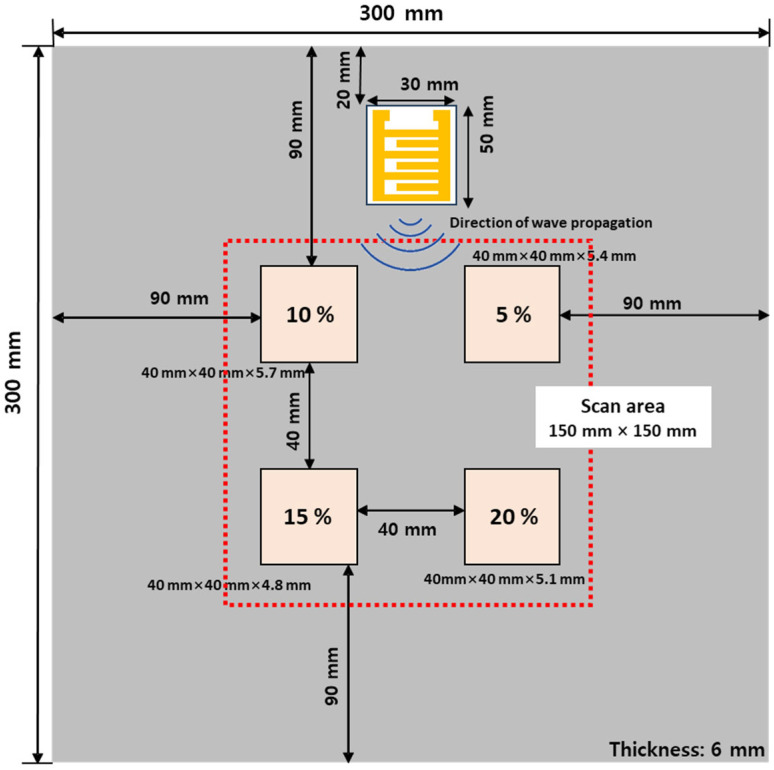
Carbon steel specimen showing defects at four different depths.

The tested material was carbon steel with a thickness of 6 mm, featuring four different depths of fabricated defects. These defects were square-shaped, measuring 40 mm by 40 mm, and were located at depths of 0.3, 0.6, 0.9, and 1.2 mm, as illustrated in Figure 10. To conduct the experiment, the scan area was set to 150 mm × 150 mm in relation to the defect.

The materials of the test piece, defect size, etc., were set to the same configuration as the existing test piece to perform a comparative analysis with the results of previous research. Such defects correspond to those outlined in the KEPIC 3521 standards for nuclear power plants [36,37,38,39].

### 3.3. Experimental Results

**Figure 11:**
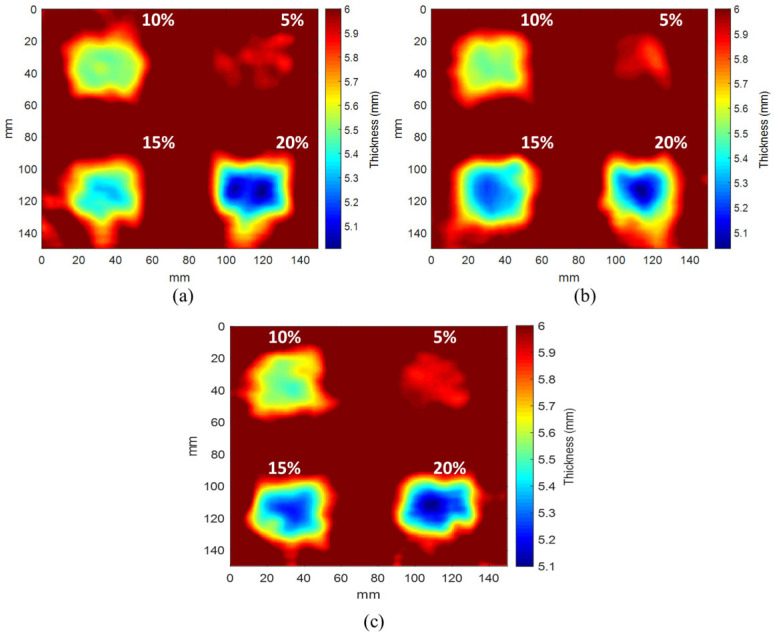
Comparative results for three SLDV techniques: (**a**) IDT-based SLDV, (**b**) dry-coupled IDT-based SLDV, and (**c**) low-power IDT-based SLDV.

This section presents the results of applying the $d_{33}$-type CSL2 IDT in both low-power IDT-based and dry-coupled IDT-based SLDV configurations. Figure 11 displays the experimental imaging results for the three SLDV techniques; panels (a) and (b) show the results of previous studies, and panel (c) displays the results of the low-power IDT-based SLDV.

All three techniques resulted in favorable outcomes; even the 20% depth defect was imaged with greater clarity than the other defects. This is attributable to the difference in wavenumber between the defect and sound regions. As illustrated in Figure 4, the wavenumber sensitivity was high at 6 mm, and the error rate was minimal because of the proportional relationship between the accuracy and wavenumber sensitivity. 

**Figure 12:**
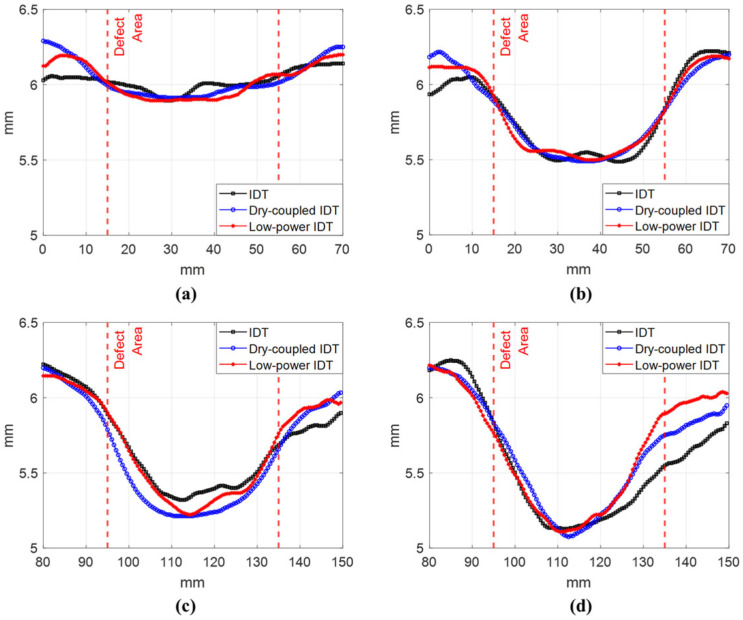
Thickness measurement results of three SLDV techniques for (**a**) 5%, (**b**) 10%, (**c**) 15%, and (**d**) 20% depth defects.

Figure 12 displays the section thickness measurements along the *y*-axis for each defect depicted in Figure 11. The figure compares the performance of each SLDV technique in terms of the defects.

As illustrated in Figure 11, the results of the low-powered IDT-based SLDV method were comparable to those of the previously investigated IDT and dry-coupled IDT. This substantiates the efficacy of the low-powered IDT-based SLDV technique. The results of Figure 11 and Figure 12 are summarized in Table 2.

The results presented in Figure 11 and Figure 12, as well as in Table 2, demonstrate the enhanced performance of the low-power IDT SLDV technique in comparison to that of the other methods. It is evident that a 5% depth defect is associated with an elevated error rate, exceeding 65%. Furthermore, determining the thickness of thin plates with precision is a challenging task. The most effective outcomes were observed in instances where the depth defects were present at a rate of 10–15%. The low-power IDT-based SLDV demonstrated its effectiveness in accurately representing all the tested defect depths, underscoring its potential as a viable tool for detecting defects in carbon steel specimens.

## 4. Conclusions

In this study, a PMN-PZT [Pb(Mg_1/3_Nb_2/3_)O_3_-Pb(Zr,Ti)O_3_] single-crystal (product code: CSL2) low-power IDT sensor was successfully developed. A notable advantage of the SSCG method is the absence of composition gradients within the resulting single crystal, which ensures chemical uniformity. This uniformity is attributed to the fact that the PMN-PZT phase does not melt during the fabrication process, thereby preserving the integrity of the single crystal structure. Based on a PMN-PZT, the sensor was designed for SLDV applications that do not need a power amplifier. CSL2 exhibited a higher piezoelectric charge constant compared to that of APC 850 (PZT) and functioned effectively without requiring high excitation voltages. This feature facilitated the efficient low-power detection of defects, marking a significant advance in the field of IDT sensor technology. The sensor, using the unique properties of PMN-PZT, exhibited an enhanced performance relative to both the traditional IDT-based SLDV system and those using the dry-coupled IDT configuration. The improvement was attributed to the inherent advantages offered by the CSL2 sensor’s design and material composition. Experiments were conducted with four different depth specimens. In the experiments, high wavenumber sensitivity led to a low error rate, and detecting a 5% defect was more difficult than detecting a 20% defect due to the low wavenumber sensitivity of the 5% defect. These results are consistent with the results of previous papers [36,37,38,39]. The low-power IDT-based SLDV technology is anticipated to have a significant impact on non-destructive testing and evaluation, as it offers a promising solution for industrial applications in which low-power operation is essential. This study opens new avenues for efficiently and effectively monitoring structural integrity in a variety of settings, contributing to safer and more sustainable industrial practices.

## Figures and Tables

**Table 1:** Specifications of CSL2 and APC 850.

	Type	CSL2 (001)	CSL2 (011)	APC 850
Parameter				
tanδ		0.011	0.013	≤2.00
$k_{33}$		0.91	0.88	0.72
$k_{31}$		0.45	0.76	0.36
$d_{33}$ (pC/N)		1952	753	400
$-d_{31}$ (pC/N)		750	919	175

**Table 2:** Summary of experimental SLDV results.

Depth	IDT-Based SLDV	Dry-Coupled IDT-Based SLDV	Low-Power IDT-Based SLDV
5% (Error rate) (0.3 mm)	1.77% (64.6%) (0.11 mm)	1.47% (70.6%) (0.09 mm)	1.7% (66%) (0.10 mm)
10% (Error rate) (0.6 mm)	7.52% (24.8%) (0.45 mm)	8.5% (15%) (0.51 mm)	8.37% (16.3%) (0.50 mm)
15% (Error rate) (0.9 mm)	11.17% (25.5%) (0.67 mm)	13.17% (12.2%) (0.79 mm)	13% (13.3%) (0. 78 mm)
20% (Error rate) (1.2 mm)	14.5% (27.5%) (0.87 mm)	15.5% (22.5%) (0.93 mm)	14.83% (25.8%) (0.89 mm)

## Data Availability

The data that support the findings of this study are available from the corresponding author upon reasonable request.
